# Double-Convex Peroneal Tubercle Morphology and MRI-Detected Peroneal Tendon Abnormality in a Non-Lateral Referral Cohort

**DOI:** 10.3390/diagnostics16081184

**Published:** 2026-04-16

**Authors:** Volkan Gür, Mehmet Burak Gökgöz, Abdurrahman Aydın, Ahmet Issın, Muhammet Ali Can, Oğuzhan Yanmaz, Nizamettin Koçkara, Furkan Yapıcı

**Affiliations:** 1 Department of Orthopedics and Traumatology, Faculty of Medicine, Erzincan Binali Yıldırım University, 24100 Erzincan, Türkiye; drvolkangur@hotmail.com (V.G.); dr.m.burakgokgoz@gmail.com (M.B.G.); canmuhammetali93@gmail.com (M.A.C.); oguzhanyanmaz@hotmail.com (O.Y.); nzmttn@yahoo.com (N.K.); 2 Department of Orthopedics and Traumatology, Düzce Çağsu Hospital, 81600 Düzce, Türkiye; draaydin7@gmail.com; 3 Department of Orthopedics and Traumatology, Faculty of Medicine, Çanakkale Onsekiz Mart University, 17110 Çanakkale, Türkiye; ahmet.issin@gmail.com

**Keywords:** peroneal tubercle, tubercle morphology, single-convex morphology, double-convex morphology, peroneal tubercle height, anatomical variation, ankle MRI, peroneal tendon abnormality, tenosynovitis

## Abstract

**Background**: The peroneal tubercle demonstrates substantial morphologic variability and may influence peroneal tendon mechanics. This study evaluated whether peroneal tubercle morphology and size are associated with MRI-detected peroneal tendon abnormality in patients undergoing ankle MRI for ankle pain without documented lateral malleolar/retromalleolar or peroneal tendon-specific symptoms. **Methods**: In this retrospective cross-sectional study, 487 ankle MRI examinations obtained between 2020 and 2023 were analyzed after excluding cases with lateral ankle/peroneal symptoms, clinically significant acute trauma, prior ankle surgery, or systemic inflammatory disease. Two orthopedic surgeons independently classified peroneal tubercle morphology (single-convex vs. double-convex) and measured tubercle height and anteroposterior length. Peroneal tendon abnormality was defined as MRI features consistent with tendinopathy and/or tenosynovitis. Inter- and intraobserver reliability were assessed using intraclass correlation coefficients and Cohen’s kappa. Unadjusted associations were assessed using Fisher’s exact test and point-biserial correlation. Multivariable logistic regression was performed to evaluate independent associations after adjustment for age, sex, BMI, and hindfoot alignment. **Results**: Mean age was 43.6 ± 15.6 years; 226 participants were male, and 261 were female. Peroneal tendon abnormality was present in 227/487 examinations (46.6%). Double-convex morphology showed a higher prevalence of abnormality than single-convex morphology (69.6% vs. 43.6%; unadjusted OR 2.97, 95% CI 1.63–5.41; *p* < 0.001). In the adjusted model, double-convex morphology remained independently associated with peroneal tendon abnormality (OR 2.85, 95% CI 1.52–5.34; *p* = 0.001). Tubercle height showed a modest independent association (OR 1.12 per mm, 95% CI 1.04–1.20; *p* = 0.002), whereas tubercle length was not associated (OR 1.008, 95% CI 0.981–1.036; *p* = 0.541). Reliability was excellent for tubercle height, tubercle length, and hindfoot alignment measurements and substantial to excellent for categorical ratings. **Conclusions**: In this non-lateral referral cohort, double-convex peroneal tubercle morphology was independently associated with higher odds of MRI-detected peroneal tendon abnormality. These findings reflect cross-sectional imaging associations rather than causation and should be interpreted with caution, given the heterogeneous MRI endpoint and the routine clinical MRI protocol.

## 1. Introduction

The peroneal tendons (peroneus longus and peroneus brevis) are key dynamic stabilizers of the lateral ankle and hindfoot, functioning as primary evertors and contributing to control of hindfoot varus/valgus and rotational stability during gait. Disorders of the peroneal tendons—including tendinopathy/tenosynovitis, longitudinal split tears, and (sub)luxation—represent a clinically important but frequently under-recognized source of lateral ankle or hindfoot pain, and they may coexist with chronic lateral ankle instability or other structural abnormalities [1]. Importantly, the “lateral ankle complex” is anatomically and biomechanically heterogeneous: multiple bony and soft-tissue variants have been proposed to influence tendon course, contact pressures, and sheath/retinacular constraints, thereby potentially contributing to peroneal tendon abnormalities rather than any single factor acting in isolation [1]. Recent literature continues to emphasize that peroneal tendon disorders are frequently underrecognized and often coexist with chronic lateral ankle instability, cavovarus alignment, and other lateral ankle abnormalities [2,3].

The peroneal tubercle (also referred to as the peroneal trochlea or trochlear process) is a bony prominence on the lateral wall of the calcaneus that separates the peroneus brevis (superior) and peroneus longus (inferior) tendons as they course posterior to the lateral malleolus and distally along the lateral calcaneus [4,5,6,7,8,9]. Considerable inter-individual variability exists in the presence, dimensions, and configuration of the tubercle, and reported prevalence varies substantially depending on the population studied and the assessment method. In an osteological series, Hyer et al. described a peroneal tubercle prevalence of approximately 90% and proposed a morphology-based classification (e.g., flat, prominent, concave/tunnel-like variants), emphasizing potential relevance to peroneus longus tendon pathology [4]. In contrast, imaging-based studies have reported lower detection rates, likely reflecting differences in imaging technique, definition thresholds, and the inherent difficulty of delineating small cortical prominences. For example, Taneja et al. reported frequent absence of an identifiable peroneal tubercle on ankle MRI and demonstrated that the measured tubercle size varies widely among individuals [5]. Additional CT- and MRI-based investigations likewise report variable prevalence and dimensions of the tubercle in both adult and pediatric populations [6,7,8,9,10]. Collectively, these studies indicate that “normal” tubercle morphology spans a broad spectrum, and the simple dichotomy of “present/absent” is insufficient to capture clinically relevant phenotypes. More recent imaging-based studies further suggest that lateral ankle anatomic variants are common and should not automatically be interpreted as independent pathologic findings in the absence of compatible clinical correlation [11,12,13].

Beyond size, the geometric configuration of the peroneal tubercle may be important because shape could plausibly influence tendon curvature, focal contact pressure, and the available space within the peroneal tendon sheath. Contemporary imaging classifications have therefore attempted to describe reproducible morphologic patterns. Vosoughi et al., using CT-based assessment, categorized the tubercle into distinct configurations (e.g., single-convex and double-convex patterns among others) and highlighted that a single “hypertrophy” cut-off could be misleading if morphology is not considered [10]. This concept is clinically relevant because an enlarged or prominent tubercle has historically been implicated in stenosing peroneal tenosynovitis and tendon attrition, particularly involving the peroneus longus beneath the tubercle [4]. In an MRI-based cohort, Taneja et al. demonstrated that patients with inframalleolar peroneal tendon abnormalities had a significantly larger peroneal tubercle and proposed an MRI-derived cut-off with moderate diagnostic performance for partial tears [5]. However, the emphasis of most prior work has been on tubercle height/size rather than on routinely applicable morphology-focused metrics, and the incremental value of tubercle shape on standard ankle MRI remains incompletely defined.

Interpretation of peroneal tendon abnormalities on MRI is further complicated by two practical considerations. First, MRI-detected peroneal tendon signal alterations and sheath fluid can be encountered even in individuals without lateral ankle symptoms, raising the possibility that at least some findings are non-lateral referral relative to the presenting complaint rather than clearly symptomatic disease. Saupe et al. prospectively demonstrated that anatomic variants traditionally thought to be associated with peroneal tendon disorders—including variations in the retromalleolar groove shape and peroneal tubercle size—can be observed in volunteers with asymptomatic ankles [9]. Similarly, Galli et al. reported that several lateral ankle anatomic variants and peroneal imaging findings may be present in patients without a history of lateral ankle pain or trauma, cautioning against assuming clinical significance based on MRI appearance alone [14]. Second, technical factors and artifacts may contribute to apparent intratendinous signal changes. Prior work has shown that the magic-angle phenomenon and related sequence/positioning effects can increase signal within ankle tendons on routine MRI, potentially mimicking tendinopathy or partial tearing and thereby increasing the risk of over-interpretation if clinical correlation is absent [15,16]. These issues reinforce the importance of symptom-directed interpretation and highlight the potential utility of objective anatomic context—such as peroneal tubercle morphology—when radiologists and clinicians encounter peroneal tendon signal changes on ankle MRI performed for non-lateral complaints. Additional recent studies indicate that space-occupying variants within the peroneal compartment, particularly the peroneus quartus and distally extended or hypertrophic peroneus brevis muscle belly, may contribute to tendon crowding, instability, or MRI-detected abnormality [17,18,19].

Within this framework, an imaging association study focused on patients undergoing ankle MRI for ankle pain but without documented lateral malleolar/peroneal tendon symptoms has a distinct purpose: to clarify whether specific peroneal tubercle morphologies are associated with MRI-detected peroneal tendon abnormalities in a non-lateral referral cohort. Such information may improve consistency in MRI interpretation when peroneal tendon findings are identified outside the primary clinical pain location. Therefore, the primary aim of this study was to evaluate the association of peroneal tubercle morphology, height, and anteroposterior length with MRI-detected peroneal tendon abnormality on ankle MRI in patients without documented lateral ankle/peroneal tendon symptoms. We hypothesized that certain morphologic patterns of the peroneal tubercle—specifically single-convex versus double-convex configurations—would demonstrate different frequencies of MRI-detected peroneal tendon abnormality.

## 2. Materials and Methods

### 2.1. Study Design

This was a retrospective, cross-sectional imaging association study conducted at the Orthopedics and Traumatology Clinic at Erzincan Binali Yıldırım University. The study was performed in accordance with the Declaration of Helsinki and was approved by the Clinical Research Ethics Committee of Atatürk University Faculty of Medicine (Decision No: B.30.2.ATA.0.01.00/423; Date: 2 June 2022). The primary objective was to evaluate the association of peroneal tubercle morphology, height, and anteroposterior length with MRI-detected peroneal tendon abnormality in patients undergoing ankle MRI for ankle pain without lateral ankle/peroneal tendon symptoms.

### 2.2. Participants

A total of 487 ankle MRI examinations obtained between 2020 and 2023 from patients aged 18–65 years were included. All MRI examinations were performed due to ankle pain.

**Figure 1:**
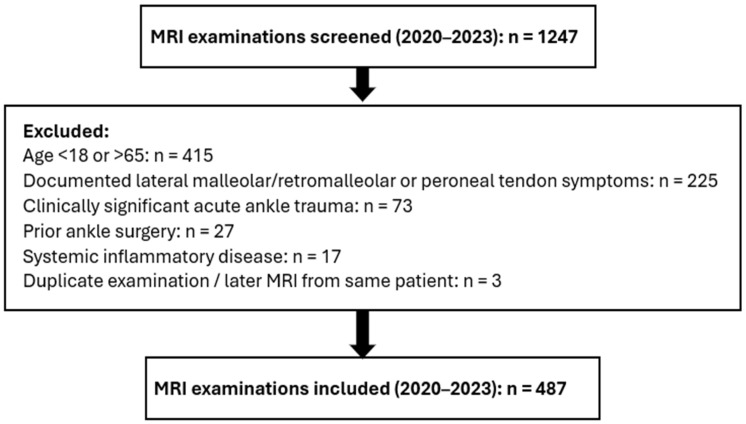
Flowchart of patient selection and exclusion for the final MRI cohort.

To minimize inclusion of patients in whom peroneal tendon findings might be symptom-driven, patients were included only if clinical documentation at the time of MRI referral did not describe lateral malleolar/retromalleolar pain or symptoms suggestive of peroneal tendon disorders (e.g., focal tenderness along the peroneal tendons, lateral swelling, snapping, or pain specifically attributed to the peroneal tendons). Patients were excluded if records documented clinically significant acute ankle trauma (e.g., fracture, dislocation, or an ankle sprain requiring medical evaluation), prior ankle surgery, or systemic inflammatory disease. Because minor remote ankle sprains are common and may not be consistently documented in retrospective records, a history of minor sprain cannot be fully excluded. Figure 1 presents the flowchart for patient selection and exclusion in the final MRI cohort.

Pain location and/or the primary MRI indication were abstracted retrospectively from orthopedic outpatient notes and/or MRI requisition forms and categorized a priori as medial ankle pain, anterior ankle pain, posterior hindfoot/Achilles-related pain, or diffuse/unspecified ankle pain when no focal location was documented. Demographic variables (age, sex, and laterality) were recorded. Body mass index (BMI) recorded closest to the MRI date was retrospectively extracted from the electronic medical record. Hindfoot alignment was assessed on MRI as described below and was included, together with age, sex, and BMI, as a covariate in the multivariable analysis. Because of the retrospective design, other clinically relevant factors—such as physical activity level, chronic ligamentous instability, and anatomic variants within the peroneal compartment (e.g., peroneus quartus, low-lying peroneus brevis muscle belly, and os peroneum)—were not available in a standardized manner for all examinations and were therefore not included in the adjusted model.

To avoid non-independence of observations, only one MRI examination per patient was included; if more than one eligible examination was available for the same patient during the study period, only the earliest MRI was analyzed.

### 2.3. MRI Protocol

All ankle MRI examinations were performed using a 1.5-T scanner (Symphony; Siemens Medical Solutions, Erlangen, Germany) with patients in the supine position and the ankle maintained in neutral alignment using a dedicated extremity coil. The institutional protocol included T1-weighted spin-echo sequences in coronal, sagittal, and axial (oblique-axial) planes and T2-weighted fast spin-echo sequences in coronal and axial planes (without fat suppression, per institutional protocol). Axial (oblique-axial) sequences were acquired with a slice thickness of 3.0 mm and an interslice gap of 0.3 mm.

Because the examinations were obtained using a routine clinical ankle MRI protocol rather than a protocol optimized specifically for subtle peroneal tendon pathology, the potential effects of field strength, slice thickness, interslice gap, and sequence selection were considered during image interpretation and are addressed further in the limitations.

### 2.4. Image Analysis

All MRI examinations were reviewed on the institutional picture archiving and communication system (PACS). Two orthopedic surgeons (V.G. and F.Y.) independently assessed (i) peroneal tubercle morphology, (ii) peroneal tubercle height, (iii) peroneal tubercle length, (iv) MRI-based hindfoot alignment, and (v) MRI signs of peroneal tendinopathy/tenosynovitis using predefined imaging criteria. Readers were blinded to each other’s measurements.

After completion of the independent reviews, discrepant categorical classifications were re-evaluated in a structured consensus session with simultaneous image review. If consensus could not be achieved, a third senior reviewer (N.K.) adjudicated the final classification. The final consensus/adjudicated dataset was used for the main association analyses. Reliability analyses were calculated using the first-round independent readings (prior to consensus/adjudication).

For continuous measurements (tubercle height, tubercle length, and hindfoot alignment), each reader performed measurements independently for all examinations; the mean of the two first-round measurements was used for the primary association analyses. For categorical variables (tubercle morphology and peroneal tendon abnormality), discrepant ratings were resolved by consensus/adjudication as described above, and the final consensus/adjudicated dataset was used for association testing. Reliability analyses were based on the first-round independent readings prior to consensus/adjudication.

This reader framework was selected because the primary study objective was to evaluate reproducible morphology classification and standardized linear measurements on routine ankle MRI rather than to establish radiology-pathology correlation. Accordingly, first-round independent readings were used for reliability analysis, whereas the consensus/adjudicated dataset was used for the main association analyses to provide a single final classification for each examination.

#### 2.4.1. Peroneal Tubercle Morphology

**Figure 2:**
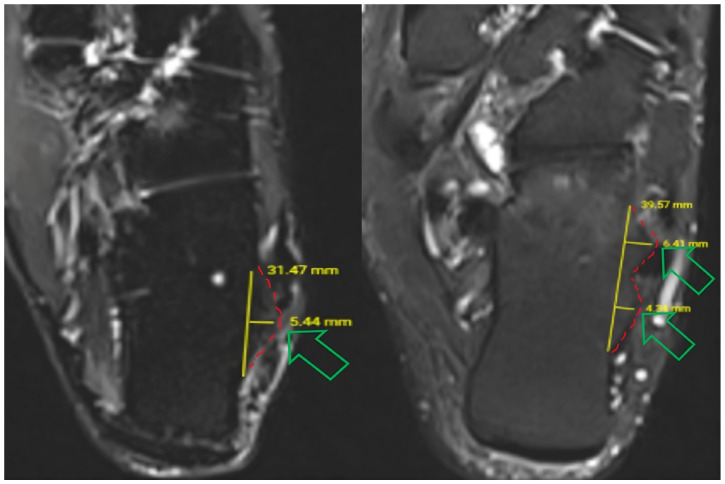
Axial MRI examples demonstrating peroneal tubercle height and anteroposterior length measurements (yellow lines) at the level of maximal prominence and the classification of single-convex (**left**) and double-convex (**right**) morphologies. The green hollow arrow on the left indicates the apex of a single-convex peroneal tubercle, while the two on the right indicate the apices of a double-convex peroneal tubercle morphology. The red dashed lines mark the upper boundary of the tubercles on both the right and left.

Peroneal tubercle morphology was classified according to the framework described by Vosoughi and Tabatabaei [10]. For operational consistency on axial images, single-convex morphology was defined as a single distinct convex bony prominence along the lateral calcaneal wall. Double-convex morphology was defined as two adjacent convex prominences (anterior and posterior peaks) separated by a relative concavity/groove on the same axial level (Figure 2). In this cohort, an identifiable peroneal tubercle was present on axial MRI in all included examinations; therefore, no cases were excluded due to tubercle absence.

#### 2.4.2. Peroneal Tubercle Height Measurement

Peroneal tubercle height was measured on axial images at the level of maximal tubercle prominence, defined as the slice demonstrating the greatest protrusion from the lateral calcaneal cortex. A reference line was drawn tangential to the lateral calcaneal surface, and height was recorded as the maximum perpendicular distance from this line to the tubercle apex. Measurements were performed on both axial T1- and T2-weighted sequences when available, and the mean value was used for analysis (Figure 2). For double-convex tubercles, height was defined as the maximum perpendicular distance to the higher of the two peaks on the same axial slice.

#### 2.4.3. Peroneal Tubercle Length Measurement

Peroneal tubercle length (anteroposterior footprint) was measured on the same axial slice used for height measurement (maximal tubercle prominence). Length was defined as the maximal anteroposterior distance between the most anterior and most posterior cortical margins of the tubercle at its base along the lateral calcaneal wall (Figure 2). Measurements were performed using the PACS digital caliper tool with identical window/level settings, and the recorded value was used for descriptive and exploratory analyses.

#### 2.4.4. Hindfoot Alignment Assessment

Hindfoot alignment was assessed on coronal MRI images using a non-weight-bearing MRI-based surrogate adapted from the apparent moment arm method described by Büber et al. [20]. Because dedicated weight-bearing hindfoot radiographs were unavailable in this retrospective cohort, coronal hindfoot alignment was quantified on routine MRI rather than on standing radiographs. The tibial reference axis was defined on the coronal distal tibia, and the apparent moment arm was recorded as the signed distance between this axis and the deepest point of the calcaneus identified on the coronal series. Positive values indicated valgus alignment, whereas negative values indicated varus alignment. Measurements were performed independently by two observers using standardized digital tools on the institutional PACS workstation, and the mean of the two first-round measurements was used for analysis. Because hindfoot alignment definitions and measurement methods vary across studies, the MRI-based hindfoot alignment value used in this retrospective cohort was treated as a pragmatic surrogate covariate rather than a gold-standard assessment of coronal hindfoot alignment [21].

#### 2.4.5. MRI Criteria for Peroneal Tendon Abnormality

Peroneal tendon abnormality was assessed in the peroneus longus and/or peroneus brevis tendons, primarily using axial and coronal sequences. Peroneal tendinopathy/tenosynovitis was defined on MRI by any of the following:

(1) intratendinous increased signal on T2-weighted images consistent with tendinosis;
(2) tendon thickening compared with expected caliber; and/or
(3) tenosynovitis, characterized by fluid distention within the peroneal tendon sheath [22].

For statistical analysis, MRI features consistent with tendinopathy and/or tenosynovitis were combined into a single binary outcome (“peroneal tendon abnormality”).

### 2.5. Statistical Analysis

Statistical analyses were performed using IBM SPSS Statistics (version 20; IBM Corp., Armonk, NY, USA). Continuous variables are reported as mean ± standard deviation (SD), and categorical variables are reported as number (percentage).

Inter- and intraobserver reliability for the continuous measurements (peroneal tubercle height, peroneal tubercle length, and hindfoot alignment) was assessed using the intraclass correlation coefficient (ICC; two-way random-effects model, absolute agreement, single-measure; ICC (2,1)) with 95% confidence intervals and Bland–Altman analysis (mean bias and 95% limits of agreement). For categorical variables (tubercle morphology and peroneal tendon abnormality), agreement was assessed using Cohen’s kappa (κ) with 95% confidence intervals.

The association between peroneal tubercle height (continuous) and peroneal tendon abnormality (binary) was evaluated using point-biserial correlation (Pearson r). The association between tubercle morphology (single- vs. double-convex) and peroneal tendon abnormality was evaluated using Fisher’s exact test, and the effect size was summarized as an unadjusted odds ratio (OR) with 95% confidence interval. Pearson correlation analysis (point-biserial) was also used to evaluate the association between peroneal tubercle length and the presence of peroneal tendon abnormality. In addition, multivariable binary logistic regression was performed to evaluate independent associations with peroneal tendon abnormality. The dependent variable was the presence of peroneal tendon abnormality, and the independent variables entered into the model were tubercle morphology, tubercle height, tubercle length, age, sex, BMI, and hindfoot alignment. Hindfoot alignment was entered as a continuous variable. Adjusted ORs with 95% confidence intervals (CIs) were reported. As an exploratory additional analysis, receiver operating characteristic (ROC) curve analysis was performed using the predicted probabilities derived from the final multivariable logistic regression model. The area under the ROC curve (AUC) was calculated to assess the apparent discriminative performance of the model. Because this ROC analysis was performed in the same retrospective cohort used to derive the model, it was interpreted as exploratory rather than as a validated diagnostic performance assessment. Two-sided *p* < 0.05 was considered statistically significant. Complete data were available for all 487 examinations included in the multivariable model; therefore, no case-wise deletion was required. Hindfoot alignment was entered into the regression model as a continuous signed distance (mm), with positive values indicating valgus and negative values indicating varus.

### 2.6. Reliability Assessment

To evaluate measurement reproducibility, both intraobserver and interobserver reliability analyses were performed for peroneal tubercle height, peroneal tubercle length, and hindfoot alignment. Two observers (V.G. and F.Y.), blinded to each other’s measurements and to prior results, performed the measurements using the standardized protocols described above. Intraobserver reliability was assessed by repeat measurement after a washout interval of 4 weeks in a randomly selected subset of 100 examinations. Interobserver reliability was assessed by comparing the two observers’ measurements in the same subset.

Reliability for continuous measurements (height, length, and hindfoot alignment) was quantified using the intraclass correlation coefficient (ICC; two-way random-effects model, absolute agreement, single-measures; ICC (2,1)) with 95% confidence intervals. Agreement was further examined using Bland–Altman analysis (mean difference and 95% limits of agreement). For categorical variables (tubercle morphology and peroneal tendon abnormality), inter- and intraobserver agreement was assessed using Cohen’s kappa (κ) with 95% confidence intervals. Reliability analyses (ICC for continuous measures; κ for categorical ratings) were performed in the same randomly selected subset of 100 examinations, and intraobserver ratings were repeated after a 4-week washout period with readers blinded to their prior assessments.

## 3. Results

For peroneal tubercle height measurements (subset *n* = 100), interobserver agreement was excellent, with ICC (two-way random-effects, absolute agreement, single-measures; ICC (2,1)) = 0.972, 95% CI 0.954–0.983. Intraobserver agreement was ICC (2,1) = 0.981 (95% CI 0.968–0.989) with a 4-week washout period. Bland–Altman analysis demonstrated a mean interobserver difference of 0.003 mm, with 95% limits of agreement of −0.615 to 0.621 mm, indicating no clinically relevant systematic bias.

For peroneal tubercle length measurements (subset *n* = 100), interobserver agreement was excellent, with ICC (2,1) = 0.989, 95% CI 0.982–0.994. Intraobserver agreement was ICC (2,1) = 0.981, 95% CI 0.968–0.989. Bland–Altman analysis demonstrated a mean interobserver difference of 0.105 mm with 95% limits of agreement from −1.805 to 2.016 mm.

For hindfoot alignment measurements (subset *n* = 100), the mean value was 5.4 ± 6.4 mm. Interobserver agreement was excellent, with ICC (2,1) = 0.960 (95% CI 0.940–0.974). Intraobserver agreement was also excellent for both observers, with ICC (2,1) = 0.962 (95% CI 0.942–0.975) and ICC (2,1) = 0.958 (95% CI 0.936–0.973). Bland–Altman analysis showed a mean interobserver difference of 0.08 mm, with 95% limits of agreement from −2.60 to 2.76 mm, indicating no clinically relevant systematic bias.

In the reliability subset (*n* = 100), interobserver agreement for categorical assessments was κ = 0.88 (95% CI 0.81–0.94) for peroneal tubercle morphology and κ = 0.84 (95% CI 0.76–0.91) for peroneal tendon abnormality. Intraobserver agreement was κ = 0.92 (95% CI 0.87–0.96) for morphology and κ = 0.89 (95% CI 0.83–0.94) for tendon abnormality.

MRI-detected peroneal tendon abnormality was present in 227 of 487 examinations (46.6%). Among examinations with MRI-detected peroneal tendon abnormality, 39 (17.2%) exhibited double-convex peroneal tubercle morphology and 188 (82.8%) exhibited single-convex morphology. Among examinations without peroneal tendon abnormality, 17 exhibited double-convex morphology and 243 exhibited single-convex morphology. A statistically significant but weak association was observed between peroneal tubercle height and peroneal tendon abnormality (r = 0.166, *p* < 0.001). Double-convex morphology was associated with higher odds of peroneal tendon abnormality compared with single-convex morphology (unadjusted OR 2.97, 95% CI 1.63–5.41; Fisher’s exact test *p* < 0.001).

In multivariable logistic regression analysis including peroneal tubercle morphology, height, and length, and adjusted for age, sex, BMI, and hindfoot alignment, double-convex morphology remained independently associated with peroneal tendon abnormality (OR 2.85, 95% CI 1.52–5.34, *p* = 0.001). Tubercle height also demonstrated a modest independent association (OR 1.12, 95% CI 1.04–1.20, *p* = 0.002), whereas tubercle length was not significantly associated (OR 1.008, 95% CI 0.981–1.036, *p* = 0.541). Among the covariates, age showed a weak but statistically significant association, whereas sex, BMI, and hindfoot alignment were not significantly associated with the outcome. Descriptive and unadjusted associations are presented in Table 1, and adjusted multivariable estimates are presented in Table 2.

**Figure 3:**
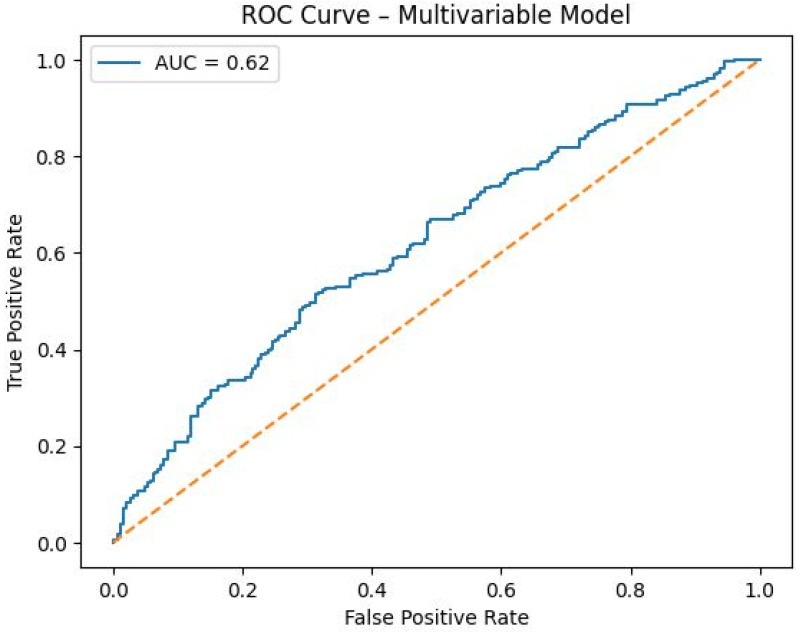
Receiver operating characteristic (ROC) curve of the final multivariable logistic regression model, including peroneal tubercle morphology, height, length, age, sex, BMI, and hindfoot alignment. The area under the curve (AUC) was 0.62, indicating limited discriminative performance.

Exploratory ROC curve analysis based on the predicted probabilities of the final multivariable logistic regression model demonstrated limited discriminative performance, with an area under the curve (AUC) of 0.62 (Figure 3). Thus, although double-convex morphology and tubercle height remained independently associated with peroneal tendon abnormality, the overall classification performance of the combined model was modest.

## 4. Discussion

In this retrospective cross-sectional MRI association study of patients who underwent ankle MRI for non-lateral ankle complaints, we found that double-convex peroneal tubercle morphology was associated with a higher prevalence of MRI-detected peroneal tendon abnormality than single-convex morphology on unadjusted analysis, and that this association persisted after adjustment for age, sex, BMI, and hindfoot alignment. Tubercle height also showed a modest independent association, whereas tubercle length did not. Taken together, these findings suggest that tubercle configuration may represent an anatomic correlate of peroneal tendon signal alteration on MRI, even when the peroneal tendons are not the primary documented source of symptoms.

Peroneal tendon disorders are widely regarded as an under-recognized cause of lateral hindfoot pain and dysfunction, and they frequently coexist with other lateral ankle pathology, including ligamentous instability and hindfoot deformity [1,23,24]. Beyond acquired factors, multiple anatomic variants have been described that may contribute to lateral ankle symptoms or peroneal tendon pathology, including hypertrophy or prominence of the peroneal tubercle, a low-lying peroneus brevis muscle belly, an accessory peroneus quartus muscle, variation in the retromalleolar fibular groove, and the presence of an os peroneum [23,24]. Importantly, not all imaging abnormalities correlate with clinical symptoms, and non-lateral referral findings are common in foot and ankle MRI datasets obtained for other reasons [25,26,27,28,29,30]. Our study adds to this literature by focusing on peroneal tubercle morphology in a cohort where lateral symptoms were specifically excluded, thereby evaluating peroneal tendon findings in a non-lateral referral setting.

A central methodological point is that our cohort was not globally asymptomatic: patients had ankle symptoms prompting MRI; however, patients with lateral malleolar pain, clinical suspicion of peroneal tendon disease, or lateral ankle symptoms were excluded. Therefore, the term “non-lateral referral” in this manuscript refers to peroneal tendon abnormalities detected on MRI that were not concordant with the documented clinical pain location/suspected diagnosis at the time of imaging. This distinction matters because multiple studies have shown that peroneal tendon abnormalities can be detected on routine MRI even in the absence of clinically apparent peroneal tendon symptoms. For example, O’Neil et al. reported that a substantial proportion of MRIs from individuals without documented lateral ankle trauma demonstrated peroneal tendon abnormalities, emphasizing the need for clinical correlation when interpreting isolated peroneal MRI findings [25]. Similarly, in cohorts investigating alignment, cavus and hindfoot varus have been associated with higher rates of MRI-detected peroneal tendon pathology, yet symptom status may not differ substantially among alignment groups, again underscoring that imaging abnormalities do not automatically imply symptomatic disease [30]. Accordingly, our results should be interpreted as an imaging association rather than a demonstration of symptomatic peroneal tendon disease or a basis for prophylactic intervention. Recent studies comparing MRI and ultrasound with surgical or intra-operative findings show that diagnostic performance varies by lesion type and that routine imaging retains a meaningful risk of both undercalling and overcalling peroneal pathology [31,32,33,34,35,36].

The peroneal tubercle serves as a bony landmark on the lateral calcaneus and functions as a pulley/separator for the peroneal tendons. Hypertrophy or prominence of this structure has long been discussed as a potential contributor to peroneal tendon irritation or tenosynovitis, particularly for the peroneus longus as it courses inferior to the tubercle [24]. Clinical case reports and small series have described stenosing tenosynovitis or tendon injury in the setting of a prominent/hypertrophic tubercle, and surgical resection is typically discussed only when symptoms persist despite conservative management [24]. However, the broader clinical literature is dominated by symptomatic cases, whereas the relationship between tubercle morphology and MRI findings in patients without lateral symptoms is less well characterized.

Our findings partly align with the conceptual framework that increased bony prominence (or a configuration that increases mechanical contact) may relate to tendon signal alterations; however, our study differs from symptom-driven series by evaluating a cohort where lateral symptoms were excluded. In addition, our study emphasizes morphology (single- versus double-convex) rather than using height alone. This is relevant because peroneal tubercle–tendon interactions may be influenced by contour and “edge geometry,” not merely by maximal height. The observation that tubercle height showed only a weak association with MRI findings also suggests that morphology and local geometry may provide information beyond a single linear dimension.

Importantly, when morphology, height, and length were evaluated together in a multivariable model and adjusted for age, sex, BMI, and hindfoot alignment, double-convex morphology remained independently associated with peroneal tendon abnormality, whereas tubercle height showed a smaller but still significant association and tubercle length remained non-significant. Exploratory ROC analysis further showed that the overall model had only limited discriminative performance (AUC = 0.62). Together, these findings suggest that tubercle configuration may be more relevant than simple linear dimensions in contextualizing MRI-detected tendon abnormality, but they do not support the use of these variables as standalone diagnostic classifiers.

From a biomechanical perspective, a double-convex tubercle may alter tendon excursion by increasing local curvature, creating two adjacent contact peaks rather than a single smooth contour, and thereby changing focal pressure distribution within the peroneal compartment. Repetitive gliding across a more irregular bony interface could plausibly increase frictional loading and tenosynovial irritation, particularly when combined with conditions that already increase lateral ankle load, such as hindfoot varus/cavovarus alignment or chronic instability. Although our study was not designed to test these mechanisms directly, the persistence of the morphology effect after adjustment is consistent with the hypothesis that contour may matter in addition to simple tubercle prominence.

Differences from prior studies may reflect cohort selection (symptomatic versus non-lateral referral settings), imaging protocols (field strength, slice thickness, sequence selection), and outcome definitions (tenosynovitis versus tendinopathy versus tears). For instance, work comparing symptomatic patients and controls has shown that the diagnostic performance of specific MRI signs varies: intermediate signal changes may be sensitive but less specific, whereas more stringent criteria (e.g., fluid within the tendon sheath beyond certain thresholds or intermediate T2 signal changes) can be more specific for symptomatic tenosynovitis/tendinopathy [26]. Such heterogeneity in imaging criteria can influence reported associations between bony morphology and tendon “pathology” on MRI.

Although our study is observational and not mechanistic, several plausible pathways may explain why a double-convex tubercle could be associated with a higher prevalence of peroneal tendon MRI abnormalities. First, a more prominent or more sharply contoured bony prominence could increase mechanical friction or compression on the tendons during repetitive motion, potentially contributing to tenosynovial irritation or intratendinous degeneration. Second, peroneal tendon disorders are often associated with conditions that alter lateral ankle mechanics, such as chronic instability or hindfoot cavovarus alignment; these conditions can increase peroneal tendon loading and may magnify any local impingement effect from the peroneal tubercle [23,30,37]. Third, “space-occupying” variants within the peroneal compartment (e.g., peroneus quartus or a low-lying peroneus brevis muscle belly) can crowd the retromalleolar region and may contribute to tendon irritation or subluxation, potentially interacting with calcaneal morphology distally [23,24,28,29]. In clinical practice, these factors often coexist rather than acting in isolation, which is why consensus and review literature repeatedly emphasize a multifactorial model for peroneal tendon disorders [1,23,24].

A critical point in interpreting MRI-based peroneal tendon “pathology” is that signal alterations can be influenced by technical and physiologic factors. The magic angle phenomenon is particularly relevant for tendons in the ankle; Mengiardi et al. demonstrated a high prevalence of magic angle–related increased signal in several ankle tendons in the supine/neutral foot position, including the peroneal tendons, which may mimic pathology on certain sequences if not interpreted carefully and correlated with fluid-sensitive images and morphology [16]. Radiology reviews similarly emphasize pitfalls, such as the magic angle and normal variants, that can simulate disease [24].

Additionally, studies comparing MRI to intraoperative findings show that MRI can have both false positives and false negatives for peroneal tendon tears and related pathology, reinforcing that MRI findings should be interpreted within the full clinical context [27]. Therefore, although our study demonstrates an association between tubercle morphology and MRI-detected tendon abnormalities, it does not establish that all identified abnormalities represent clinically meaningful disease. This is particularly important in a cohort in which the peroneal tendons were not documented as the symptomatic focus.

The clinical relevance of our findings is interpretive rather than interventional. Specifically, recognizing a double-convex peroneal tubercle may help radiologists and clinicians contextualize peroneal tendon signal alterations detected outside the primary referral complaint on ankle MRI obtained for other complaints. In patients without lateral symptoms, isolated peroneal tendon signal abnormalities should prompt careful clinical correlation (targeted history and examination for lateral pain, instability, swelling, or snapping) rather than automatic escalation to treatment. When peroneal tendon disease is clinically suspected or becomes symptomatic, current reviews and consensus guidance support individualized, symptom-driven management—typically beginning with nonoperative measures—while reserving operative interventions for persistent, clinically concordant pathology [1,23]. Our results do not support preventive or prophylactic interventions in asymptomatic patients; rather, they support more nuanced MRI interpretation and clinical correlation when peroneal abnormalities are detected in the non-lateral referral setting.

This study has several strengths, including a relatively large MRI cohort and a design that specifically excluded lateral ankle symptoms, allowing evaluation of peroneal MRI findings in a non-lateral referral setting. However, several limitations warrant emphasis. First, because this was a retrospective referral-based MRI cohort, selection bias and limited external validity are possible; accordingly, the prevalence estimates reported here should not be generalized to asymptomatic volunteers or to surgical cohorts with clinically confirmed peroneal tendon disease. Second, although multivariable analysis was performed, residual confounding remains likely because several clinically relevant factors—including chronic ligamentous instability, activity level, and anatomic variants within the peroneal compartment—were not available in a standardized manner for all examinations and therefore could not be included in the adjusted model. Third, the MRI endpoint combined tendinopathy and/or tenosynovitis into a single binary outcome, increasing heterogeneity and potentially obscuring differences between specific tendon abnormalities; separate subcategories were not available in a sufficiently standardized manner across the full cohort to support reliable secondary subgroup analyses. Fourth, the imaging protocol reflected routine clinical practice rather than one optimized specifically for subtle peroneal tendon pathology; therefore, 1.5-T field strength, 3 mm slice thickness, interslice gap, lack of fat-suppressed T2 imaging, and artifacts such as the magic angle effect may have influenced the detection and classification of subtle tendon abnormalities [16,24,26,27,38]. Fifth, hindfoot alignment was not assessed with dedicated weight-bearing hindfoot radiographs and was instead approximated on routine non-weight-bearing coronal MRI using the apparent moment arm method described by Büber et al. [20]. Accordingly, this variable should be interpreted as an imaging-based surrogate covariate rather than as a gold-standard assessment of coronal hindfoot alignment. Prior work has shown only moderate agreement between MRI-derived apparent moment arm measurements and standing hindfoot alignment radiographs, with a tendency for MRI to yield more valgus values. Therefore, some residual misclassification of hindfoot alignment is possible. Sixth, the ROC analysis was performed in the same cohort used to derive the multivariable model and was not externally validated; accordingly, the reported AUC should be interpreted as an exploratory estimate of apparent model discrimination. Finally, because symptom location was determined retrospectively from referral documentation, the study should not be interpreted as an evaluation of clinically confirmed symptomatic peroneal tendon disease. Recent MRI literature also suggests that peroneal split lesions may be underreported on routine ankle MRI and that more advanced isotropic imaging protocols may improve lesion depiction in selected settings [31,32,34,35].

Future prospective studies should incorporate standardized clinical localization of pain, validated symptom scores, and longitudinal follow-up to clarify which MRI-detected abnormalities are clinically meaningful and whether peroneal tubercle morphology influences symptom development. Multivariable modeling incorporating hindfoot alignment, ligamentous stability, and other anatomic variants would help determine the independent contribution of tubercle morphology. Although our peroneal tubercle height measurement protocol demonstrated excellent reproducibility, future work using higher-resolution or isotropic imaging protocols and multicenter reader studies could further reduce artifact-related misclassification and improve generalizability.

In patients undergoing ankle MRI for non-lateral complaints and without documented lateral ankle symptoms, double-convex peroneal tubercle morphology was independently associated with MRI-detected peroneal tendon abnormality, while tubercle height showed a smaller association, and tubercle length showed no independent association. These findings support careful symptom-driven clinical correlation and suggest that peroneal tubercle morphology may help contextualize peroneal tendon signal alterations detected outside the primary referral complaint.

## 5. Conclusions

In patients undergoing ankle MRI for ankle pain without documented lateral malleolar or peroneal tendon-specific symptoms, double-convex peroneal tubercle morphology was independently associated with higher odds of MRI-detected peroneal tendon abnormality, whereas tubercle height showed a smaller association, and tubercle length showed no independent association. These findings should be interpreted as adjusted cross-sectional imaging associations within a non-lateral referral cohort rather than evidence of causation or clinically confirmed tendon disease.

## Figures and Tables

**Table 1:** Demographic characteristics, descriptive measurements, correlation findings, and distribution of peroneal tendon abnormality according to peroneal tubercle morphology.

Parameter	Value/Result
Total included examinations	487
Mean age (years, mean ± SD)	43.6 ± 15.6
Sex (male/female)	226/261
Affected side (right/left)	263/224
Descriptive measures	
Peroneal Tubercle Height (mm, mean ± SD)	5.54 ± 1.82
Peroneal Tubercle Length (mm, mean ± SD)	24.67 ± 6.72
Peroneal tendon abnormality present, *n* (%)	227 (46.6%)
Hindfoot alignment (mm, mean ± SD)	5.6 ± 5.8
Correlation analysis	
Height vs. tendon abnormality (Pearson *r*, *p*)	*r* = 0.166, *p* < 0.001
Length vs. tendon abnormality (Pearson *r*, *p*)	*r* = 0.03, *p* = 0.507
Tubercle morphology	
Double-convex	Positive: 39 (69.6%), Negative: 17 (30.4%), Total: 56
Single-convex	Positive: 188 (43.6%), Negative: 243 (56.4%), Total: 431
Association	
Unadjusted OR (double- vs. single-convex), 95% CI	2.97 (1.63–5.41)
*p*-value (Fisher’s exact test)	*p* < 0.001 (Significant association)

Abbreviations: SD, standard deviation; OR, odds ratio; CI, confidence interval. For hindfoot alignment, positive values indicate valgus and negative values indicate varus.

**Table 2:** Multivariable Logistic Regression Analysis.

Variable	OR	95% CI	*p* Value
Double-convex morphology	2.85	1.52–5.34	0.001
Height (mm)	1.12	1.04–1.20	0.002
Length (mm)	1.008	0.981–1.036	0.541
Age (years)	1.013	1.001–1.025	0.031
Sex (Male = 1)	0.862	0.602–1.234	0.418
BMI (kg/m^2^)	0.972	0.931–1.014	0.188
Hindfoot alignment (mm)	0.997	0.966–1.029	0.849

Abbreviations: OR, odds ratio; CI, confidence interval; BMI, body mass index.

## Data Availability

The data supporting the findings of this study are available from the corresponding author upon reasonable request. Due to ethical and legal restrictions, the data are not publicly available.

## Abbreviations

CT	Computed tomography
MRI	Magnetic resonance imaging
PACS	Picture archiving and communication system
